# Feasibility of Targeting Traf2-and-Nck-Interacting Kinase in Synovial Sarcoma

**DOI:** 10.3390/cancers12051258

**Published:** 2020-05-16

**Authors:** Tetsuya Sekita, Tesshi Yamada, Eisuke Kobayashi, Akihiko Yoshida, Toru Hirozane, Akira Kawai, Yuko Uno, Hideki Moriyama, Masaaki Sawa, Yuichi Nagakawa, Akihiko Tsuchida, Morio Matsumoto, Masaya Nakamura, Robert Nakayama, Mari Masuda

**Affiliations:** 1 Laboratory of Collaborative Research, Division of Cellular Signaling, National Cancer Center Research Institute, Tokyo 104-0045, Japan; tsekita@ncc.go.jp (T.S.); mamasuda@ncc.go.jp (M.M.); 2 Department of Orthopedic Surgery, Keio University School of Medicine, Tokyo 160-8582, Japan; t.hirozane@gmail.com (T.H.); morio@a5.keio.jp (M.M.); masa@keio.jp (M.N.); robert.a2@keio.jp (R.N.); 3 Department of Gastrointestinal and Pediatric Surgery, Tokyo Medical University, Tokyo 160-0023, Japan; ynagakawa@gmail.com (Y.N.); akihikot@tokyo-med.ac.jp (A.T.); 4 Division of Musculoskeletal Oncology, National Cancer Center Hospital, Tokyo 104-0045, Japan; ekobayas@ncc.go.jp (E.K.); akawai@ncc.go.jp (A.K.); 5 Department of Diagnostic Pathology, National Cancer Center Hospital, Tokyo 104-0045, Japan; akyoshid@ncc.go.jp; 6 Carna Biosciences, Inc., Kobe 650-0047, Japan; yuko.uno@carnabio.com (Y.U.); hideki.moriyama@carnabio.com (H.M.); masaaki.sawa@carnabio.com (M.S.)

**Keywords:** Wnt signaling, synovial sarcoma, TNIK, NCB-0846, MYC

## Abstract

Background: The treatment of patients with metastatic synovial sarcoma is still challenging, and the development of new molecular therapeutics is desirable. Dysregulation of Wnt signaling has been implicated in synovial sarcoma. Traf2-and-Nck-interacting kinase (TNIK) is an essential transcriptional co-regulator of Wnt target genes. We examined the efficacy of a small interfering RNA (siRNA) to *TNIK* and a small-molecule TNIK inhibitor, NCB-0846, for synovial sarcoma. Methods: The expression of TNIK was determined in 20 clinical samples of synovial sarcoma. The efficacy of NCB-0846 was evaluated in four synovial sarcoma cell lines and a mouse xenograft model. Results: We found that synovial sarcoma cell lines with Wnt activation were highly dependent upon the expression of *TNIK* for proliferation and survival. NCB-0846 induced apoptotic cell death in synovial sarcoma cells through blocking of Wnt target genes including *MYC*, and oral administration of NCB-846 induced regression of xenografts established by inoculation of synovial sarcoma cells. Discussion: It has become evident that activation of Wnt signaling is causatively involved in the pathogenesis of synovial sarcoma, but no molecular therapeutics targeting the pathway have been approved. This study revealed for the first time the therapeutic potential of TNIK inhibition in synovial sarcoma.

## 1. Introduction

Synovial sarcoma is a rare aggressive neoplasm that accounts for 10–20% of soft tissue sarcomas. It affects mainly adolescents and young adults [1,2], and 40–50% of patients are under the age of 30 at diagnosis [3]. The mainstay of treatment is wide surgical excision and conventional chemotherapy [4,5]. However, the disease tends to show early or late recurrence and often becomes resistant to cytotoxic agents. The 10 year disease-free survival rate of patients with distant metastases remains around 50% [6]. It is desirable to develop new molecular therapeutics targeting pathways essential for the growth and survival of synovial sarcoma. The fusion *SS18*-*SSX* (*SSX1*, *SSX2*, or *SSX4*) gene produced by a chromosomal translocation, t (X;18) (p11.2; q11.2), is detectable in ~95% of synovial sarcomas [7,8,9]. Although dysregulation of the BAF chromatin-remodeling complex has been shown to be involved in the oncogenic activity of SS18-SSX [10,11], no therapeutics that can target the product of SS18-SSX or the BAF complex have yet been developed.

The canonical (β-catenin-dependent) Wnt signaling pathway plays crucial roles in the regulation of diverse biological processes including cell proliferation, survival, migration, and polarity, specification of cell fate, and self-renewal of embryonic stem cells, and its dysregulation has been implicated in the generation and progression of various malignancies [12]. Wnt signaling is also implicated in the pathogenesis of synovial sarcoma; synovial sarcoma cells frequently show accumulation of β-catenin protein in the nucleus [13], and express Wnt target gene products such as AXIN2 (axis inhibition protein 2), DKK1 (dickkopf1), survivin, c-MYC, and cyclinD1 [14]. SS18-SSX is responsible for the nuclear translocation of β-catenin [15,16], and Wnt signaling is aberrantly activated by SS18-SSX in a transgenic mouse model; inhibition of Wnt signaling through genetic loss of β-catenin blocks synovial sarcoma tumor formation [17]. *SS18*-*SSX2*-specific small interfering RNA (siRNA) reduces the expression of Wnt target gene products [14]. Together, these studies have highlighted the Wnt signaling pathway as a potential therapeutic target for synovial sarcoma.

Through comprehensive mass spectrometry analysis of the nuclear proteins of colorectal cancer cells, we previously identified Traf2-and-Nck-interacting kinase (TNIK) as a component of the T-cell factor-4 (TCF4) and β-catenin transcriptional complex, the most downstream effector of the Wnt signaling pathway [18]. More than 80% of colorectal cancers carry inactive mutations in the *APC* tumor-suppressor gene, and Wnt signaling is activated downstream of it. We found that TNIK was essential for transactivation of Wnt target genes and that colorectal cancer cells were highly sensitive to TNIK inhibition [19,20]. We screened a compound library and identified a novel small-molecule TNIK inhibitor named NCB-0846. NCB-0846 suppresses the transcriptional co-regulator function of TNIK by modifying its conformational structure [21,22]. NCB-0846 exhibited marked anti-tumor and anti-stem-cell activities in colorectal cancer cells and patient-derived xenografts through blocking of Wnt target gene expression [21].

Based on these findings, we speculated that TNIK inhibition would be effective for treatment of synovial sarcoma. Here, we report the therapeutic potential of TNIK inhibition in synovial sarcoma.

## 2. Results

### 2.1. Activation of Wnt Signaling and TNIK in Synovial Sarcoma

**Figure 1:**
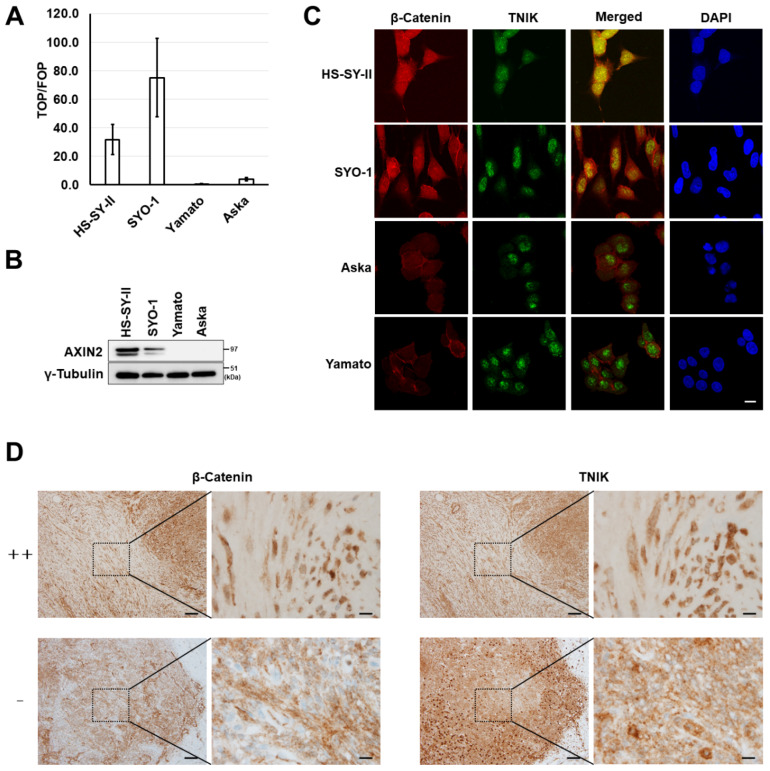
Wnt activation in synovial sarcoma. (**A**) T-cell factor (TCF)/lymphoid enhancer factor (LEF) transcriptional activity of synovial sarcoma cells. Four synovial sarcoma cell lines (HS-SY-II, SYO-1, Yamato, and Aska) were transfected with the super-TOP flash or super-FOP flash luciferase reporter, and their luciferase activity was measured 24 h later. Data represent the mean TOP/FOF ratio (± S.D.) of three replicates. (**B**) Expression of the axis inhibition protein 2 (AXIN2) and γ-tubulin (loading control) proteins determined by immunoblotting. (**C**) Dual immunofluorescence analysis of β-catenin and Traf2-and-Nck-interacting kinase (TNIK) protein expression in synovial sarcoma cells. Scale bar: 20 µm. (**D**) Immunohistochemical analysis of the β-catenin and TNIK proteins in clinical specimens of synovial sarcoma. Representative cases with strong positive (++) and negative (−) nuclear β-catenin expression are shown. Scale bars: 100 µm in low-power views (left) and 10 µm in high-power views (right).

To evaluate the activation of Wnt signaling, four synovial sarcoma cell lines were transfected with a pair of reporters (super-TOP and super-FOP luciferase reporter plasmids), and their luciferase activity was measured. Active transcription of T-cell factor (TCF)/lymphoid enhancer factor (LEF) was detected in two synovial sarcoma cell lines, HS-SY-II and SYO-1 (Figure 1A). Expression of a Wnt target gene product (AXIN2 protein) (Figure 1B) and nuclear expression of β-catenin (red, Figure 1C) were detected in these two cell lines. Nuclear translocation of TNIK is indicative of its active status [19]. Nuclear expression of TNIK was detected in all four cell lines examined (green, Figure 1C), and TNIK was co-localized with β-catenin in the nuclei of synovial sarcoma cell lines with Wnt activation (merge, Figure 1C). Using immunohistochemistry, the expression of β-catenin and TNIK was then examined in tissue specimens resected from 20 patients with synovial sarcoma. We detected nuclear staining of β-catenin in 90% (18/20) of the examined cases, and these tumors also exhibited nuclear expression of TNIK (Figure 1D and Table S1).

### 2.2. Growth Suppression of Synovial Sarcoma Cells Through Silencing of TNIK

**Figure 2:**
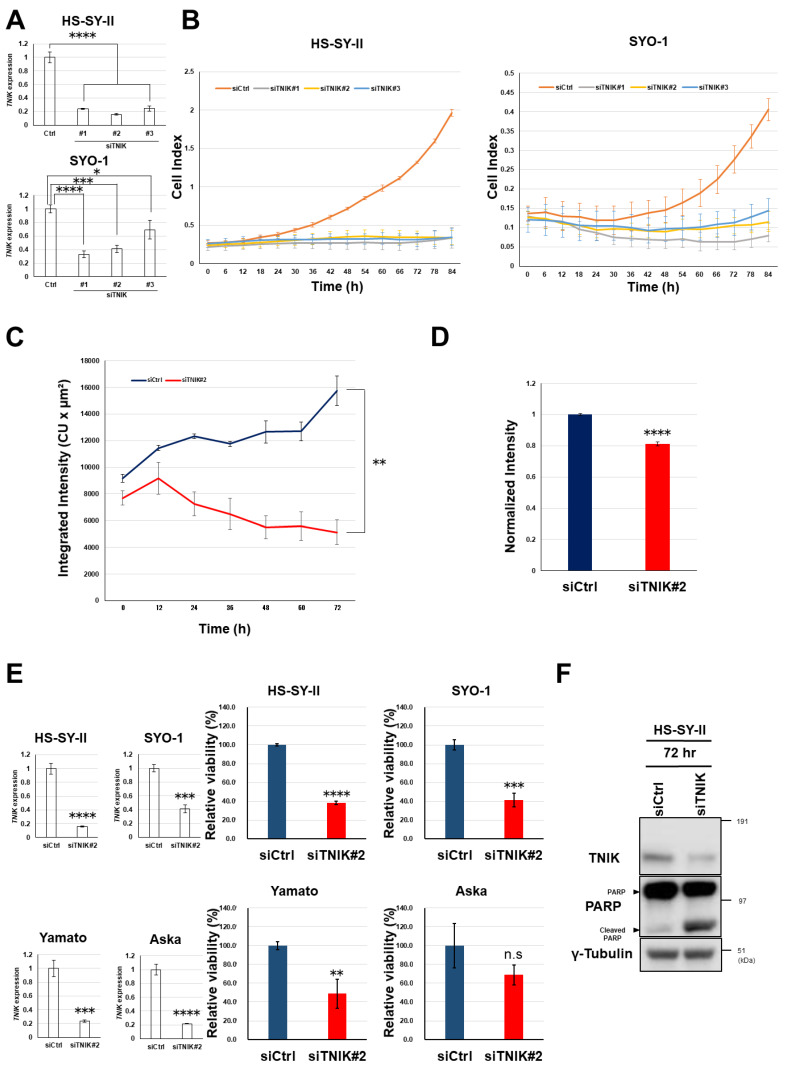
Growth suppression and apoptosis induction in synovial sarcoma cells by knockdown of *TNIK*. (**A**) HS-SY-II and SYO-1 cells were transfected with control small interfering RNA (siRNA) (siCtrl) and siRNA to *TNIK* (siTNIK#1, #2, and #3), and their relative expression of *TNIK* (normalized to *ACTB*) was quantified in triplicate by real-time RT-PCR 72 h after transfection. The expression level in cells transfected with siCtrl was set at 1. * *p* < 0.05, *** *p* < 0.0005, **** *p* < 0.0005 (multiple *t*-test corrected using the Holm–Sidak method). (**B**) Real-time growth monitoring of HS-SY-II and SYO-1 cells transfected with siCtrl and siRNA to TNIK (siTNIK#1, #2, and #3). Data represent the mean cell index (https://www.aceabio.com/products/icelligence/) ± S.D. of three replicates. (**C**,**D**) Suppression of TCF/LEF transcription by *TNIK* knockdown. HS-SY-II cells engineered to stably carry a TOP-driven green fluorescent protein (GFP) reporter were transfected with siCtrl or siTNIK#2. Average integrated intensity (summed fluorescence intensity per cell) (https://www.essenbioscience.com/media/uploads/files/8000-0193-A00_ZOOM_Fluorescence_Processing_Tech_Note.pdf#search=%27Average+Integrated+Intensity%27) was monitored every 6 h for 72 h (**C**). Total integrated intensity (total sum of fluorescence intensity per well) was normalized to ATP production 24 h after transfection (D). Data represent the mean ± S.D. of three replicates. ** *p* < 0.005, **** *p* < 0.0005 (multiple *t*-test corrected using the Holm–Sidak method). (**E**) Synovial sarcoma cells were transfected with siCtrl or siTNIK#2, and their expression of *TNIK* (normalized to *ACTB*) was quantified by real-time RT-PCR 72 h after transfection (left). Their relative viability to siCtrl (set to one) was assessed in terms of ATP production (right). ** *p* < 0.005, *** *p* < 0.0005, **** *p* < 0.0005, n.s. not significant (multiple *t*-test corrected using the Holm–Sidak method). Data represent the mean ± S.D. of three replicates. (**F**) Expression of the poly (ADP-ribose) polymerase-1 (PARP-1) and γ-tubulin (loading control) proteins determined by immunoblotting for 72 h.

Transfection of three siRNA constructs targeting *TNIK* (siTNIK#1, #2, and #3) into HS-SY-II and SYO-1 synovial sarcoma cells was confirmed to reduce the levels of *TNIK* gene expression relative to cells transfected with control siRNA (Ctrl) (Figure 2A). Real-time monitoring revealed that knockdown of *TNIK* induced the almost complete growth arrest of HS-SY-II and SYO-1 cells (Figure 2B) and significantly reduced TCF/LEF transcription in HS-SY-II cells lentivirally engineered to stably carry a TOP-driven green fluorescent protein (GFP) reporter construct (Figure 2C), even after being normalized to cell viability (Figure 2D). The four synovial sarcoma cell lines were transfected with siRNA to *TNIK* (siTNIK#2) or control siRNA (siCtrl), and their viability was assessed 72 h later. *TNIK* knockdown significantly suppressed the viability of HS-SY-II, SYO-1, and Yamato cells, but not that of Aska cells (Figure 2E). Aska cells lack Wnt activation or *MYC* gene amplification (discussed later). *TNIK* knockdown induced cleavage of poly (ADP-ribose) polymerase-1 (PARP-1) in HS-SY-II cells (Figure 2F), indicating induction of apoptosis.

### 2.3. Sensitivity of Synovial Sarcoma to NCB-0846

**Figure 3:**
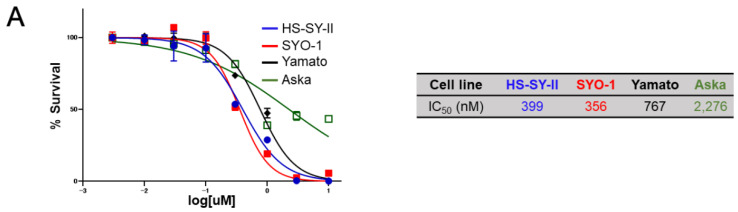

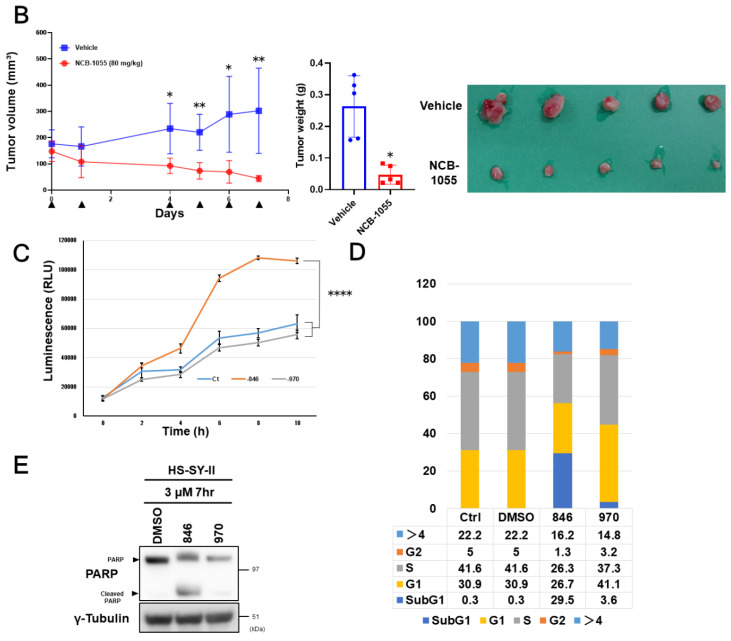
Sensitivity of synovial sarcoma to NCB-0846. (**A**) ATP production by four synovial sarcoma cell lines cultured with increasing doses of NCB-0846 for 72 h. Data represent the mean (relative to no treatment) of three replicates. (**B**) HS-SY-II cells were inoculated into the subcutaneous tissues of 6 week old female NOD.CB17-*Prkdc*^scid^/J (NOD/SCID) mice. When the average volume of the xenografts reached ~200 mm³, water (vehicle, *n* = 5) or 80 mg/kg (*n* = 5) NCB-0846 hydrochloride (NCB-1055) [21] was administered orally on the days indicated by ▼. Tumor volume was measured on the days of drug administration (left), and tumors were excised (lower right) and weighed (upper right) 7 days after the start of drug administration. * *p* < 0.05, ** *p* < 0.005 (multiple *t*-test corrected using the Holm–Sidak method). Error bars represent S.E.M. (**C**) HS-SY-II cells were cultured with dimethyl sulfoxide (DMSO) (vehicle), NCB-0846 (3 µM) or NCB-0970 (3 µM) in the presence of the Real-time-Glo™ Annexin V Apoptosis Assay Reagent (Promega), and relative luminescence unit (URL) data were collected at every 2 h over a 10 h time course. **** *p* < 0.0005 (multiple *t*-test corrected using the Holm–Sidak method). Data represent the mean of three readings for each replicate ± S.D. (**D**) HS-SY-II cells were untreated (Ctrl) or treated with DMSO, NCB-0846 (3 µM), or NCB-0970 (3 µM) for 6 h. The percentage of cells in each cell cycle fraction was determined by flow cytometry. (**E**) HS-SY-II cells were treated with DMSO (control), NCB-0846 (3 µM), or NCB-0970 (3 µM) for 7 h. The expression levels of PARP-1 and γ-tubulin (loading control) were determined by immunoblotting.

Based on the remarkable growth suppression and apoptosis induction in synovial sarcoma cells by silencing of the *TNIK* gene, the sensitivity of synovial sarcoma cell lines to a small-molecule TNIK inhibitor, NCB-0846, was then evaluated. Consistent with the siRNA to *TNIK*, NCB-0846 reduced the viability of HS-SY-II, SYO-1, and Yamato cells with a half maximal inhibitory concentration (IC_50_) of 339, 356, and 767 nM, respectively. Aska cells were insensitive to NCB-0846 and had an IC_50_ value exceeding 2.0 µM (Figure 3A). The water-soluble hydrochloride salt of NCB-0846 (named NCB-1055) [21] was administered orally to immune-deficient mice subcutaneously inoculated with HS-SY-II cells. The xenografts regressed below the baseline (before administration) even after the first administration of NCB-1055 and did not re-grow (Figure 3B). Real-time monitoring of cell-surface phosphatidylserine (PS) revealed that NCB-0846, but not its diastereomer (named NCB-0970), induced apoptotic cell death of HS-SY-II cells within 6 h after the start of drug treatment (Figure 3C). NCB-0970 was used as a negative control, i.e., a compound having the same chemical structure as NCB-0846 except for an opposite configuration of one terminal hydroxyl group [21]. An increase of the sub-G1 cell population (Figure 3D) and cleavage of PARP-1 (Figure 3E) confirmed the induction of apoptotic cell death by NCB-0846.

### 2.4. Gene Expression Profiling

**Figure 4:**
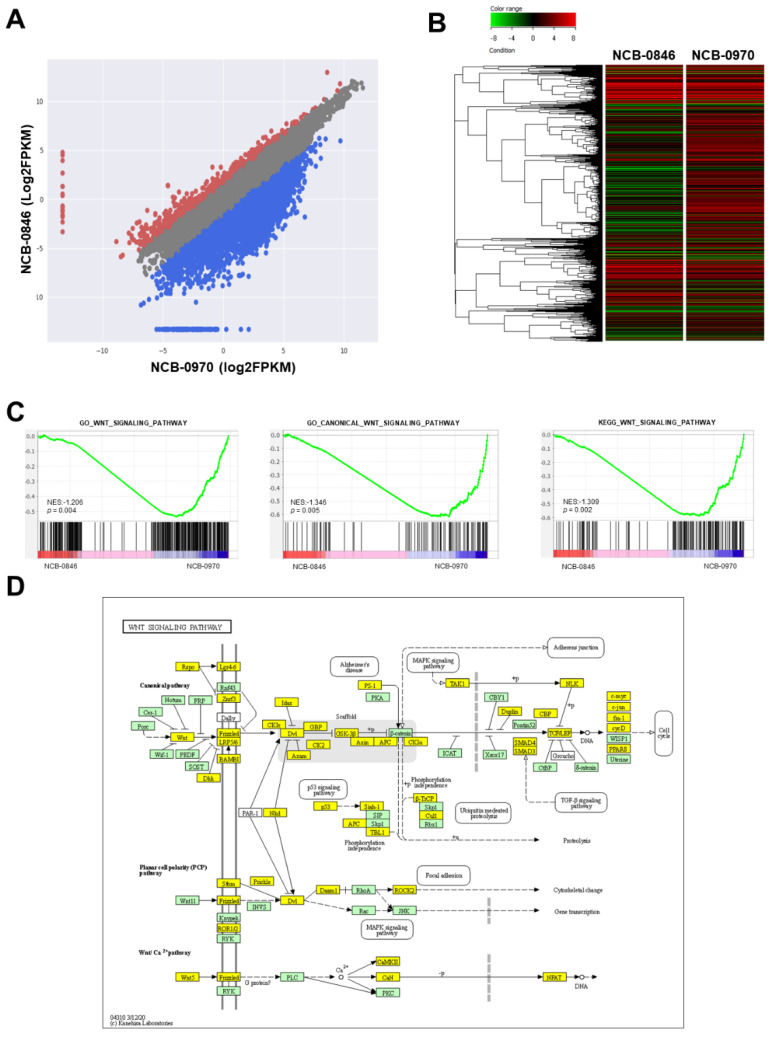
Gene expression profiling of synovial sarcoma cells treated with NCB-0846. (**A**) Scatter plot of genes differentially expressed between cells treated with NCB-0846 and NCB-0970 (negative control). Red dots represent genes upregulated more than 2-fold, and blue dots represent genes downregulated more than 2-fold in cells treated with NCB-0846. (**B**) Heat map plot of genes differentially expressed between NCB-0846 and NCB-0970. The upper color bar represents the degree of differential expression. (**C**) Gene set enrichment analysis (GSEA) showing the significant enrichment of genes annotated to the gene ontology (GO) terms “Wnt signaling pathway” (*p* = 0.004) and “canonical Wnt signaling pathway” (*p* = 0.005) and to “Wnt signaling pathway” deposited in the KEGG database (*p* = 0.002). NES: normalized enrichment score (http://software.broadinstitute.org/gsea/index.jsp). (**D**) Mapping of differentially expressed genes onto the Wnt signaling pathway. Yellow boxes indicate genes downregulated (>2-fold) by NCB-0846.

We then examined the changes in gene expression associated with the early induction of apoptosis by NCB-0846. HS-SY-II cells were exposed to NCB-0846 or NCB-0970 for 6 h, and their relative RNA expression (FPKM, fragments per kilobase of exon per million mapped reads) was determined using a next-generation sequencer. We found that the expression of a large number (6710/14,611) of genes was suppressed more than 2-fold by treatment with NCB-0846 in comparison to that with NCB-0970 (Figure 4A,B), indicating that this compound had a large impact on gene transcription beyond the suppression of Wnt target gene expression. Gene set enrichment analysis (GSEA) (Table S2) revealed significantly concordant alteration of a group of genes annotated to the Wnt signaling pathway (Figure 4C). The differentially expressed genes were mapped to the Wnt signaling pathway deposited in the Kyoto Encyclopedia of Genes and Genomes (KEGG) database (Figure 4D). We previously reported that TNIK was required for the tumor-initiating function of colorectal cancer stem cells [21,22]. Consistently, a significant proportion of downregulated genes were mapped to the signaling pathways regulating stem cell pluripotency (Figure S1). The entire RNA sequencing dataset has been deposited in the DNA Data Bank of Japan (DDBJ) Sequence Read Archive (SRA) database with the accession number DRA010051.

### 2.5. NCB-0846 Suppresses MYC Gene Expression

**Figure 5:**
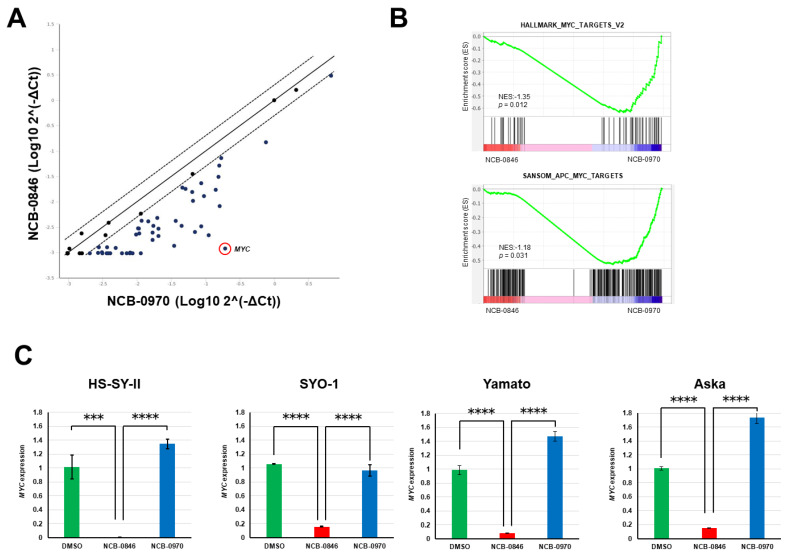
NCB-0846 suppresses *MYC* gene expression. (**A**) Comparison of Wnt target gene expression (normalized to *GAPDH* and log-transformed) of HS-SY-II cells treated with NC-0846 and NCB-0970 for 6 h. (**B**) Significant enrichment of c-MYC target genes revealed by RNA sequencing and Gene set enrichment analysis (GSEA). (**C**) Four synovial sarcoma cell lines were treated with dimethyl sulfoxide (DMSO) (control), NCB-0846 (3 μM), or NCB-0970 (3 μM) for 6 h, and expression of the *MYC* gene (relative to DMSO) was quantified by real-time RT-PCR and normalized to that of *ACTB*. *** *p* < 0.0005, **** *p* < 0.0005 (multiple *t*-test corrected using the Holm–Sidak method). Data represent the mean ± S.D. of three replicates.

Using real-time RT-PCR, we then confirmed the differential expression of Wnt target genes. The expression of 88% (78/88) of known Wnt target genes (https://web.stanford.edu/group/nusselab/cgi-bin/wnt/target_genes) was found to be downregulated (Table S3). Among these genes, *MYC* showed the most significant degree of downregulation (Figure 5A). *MYC* encodes the c-MYC protein, a transcription factor that regulates as many as 10–15% of genes in the genome [23]. We confirmed the significant enrichment of c-MYC transcriptional targets among genes regulated by NCB-0846 (Figure 5B). This marked downregulation of *MYC* was also observed in other synovial sarcoma cell lines (Figure 5C).

### 2.6. Dependency of Synovial Sarcoma Cells on MYC

**Figure 6:**
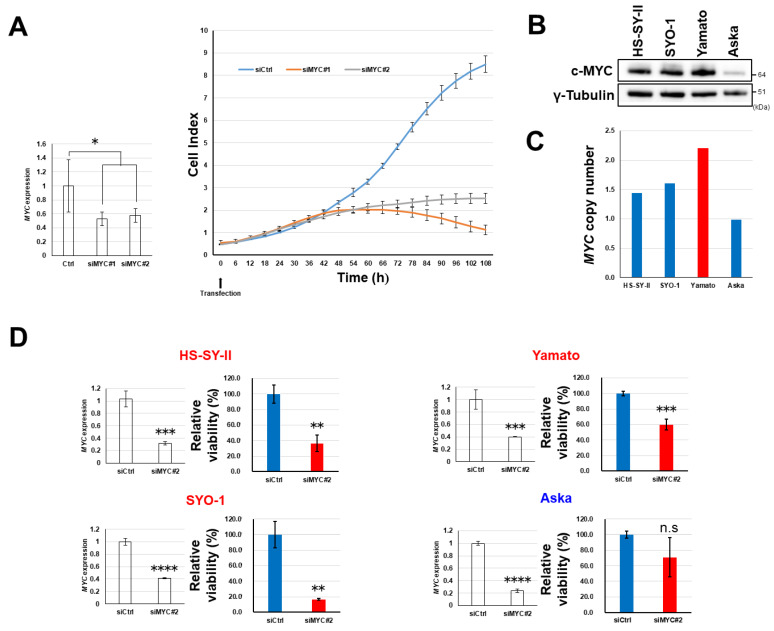
Dependence of synovial sarcoma cells on *MYC*. (**A**) Relative *MYC* expression (left) and real-time growth monitoring (right) of HS-SY-II cells transfected with control small interfering (siRNA) (siCtrl) and siRNA to *MYC* (siMYC#1 and #2). Data represent the mean *MYC* expression (normalized to *ACTB*) (left) and cell index (right) ± S.D. of three replicates. * *p* < 0.05 (multiple *t*-test corrected using the Holm–Sidak method). (**B**) The expression of c-MYC and γ-tubulin (loading control) in four synovial sarcoma cell lines was determined by immunoblotting. (**C**) Relative copy numbers of the *MYC* gene (normalized to the RNase P gene) in four synovial sarcoma cell lines determined by digital PCR. (**D**) Four synovial sarcoma cell lines were transfected with control siRNA (siCtrl) and siRNA to *MYC* (siMYC#2) in triplicate. Seventy-two hours later, their relative expression of *MYC* (normalized to *ACTB*) was quantified by real-time RT-PCR (left), and their relative viability was assessed in terms of ATP production (right). Data represent the mean ± S.D. of three replicates. ** *p* < 0.005, *** *p* < 0.0005, **** *p* < 0.0005, n.s. not significant (multiple *t*-test corrected using the Holm–Sidak method).

*MYC* is one of the targets of TCF/LEF transcription factors [24], and Wnt signaling is known to exert its oncogenic activity primarily through transactivation of the *MYC* gene [25]. We found that synovial sarcoma HS-SY-II cells with active Wnt target gene expression were highly dependent on *MYC* gene expression for proliferation (Figure 6A). However, Yamato cells also expressed the c-MYC protein (Figure 6B) in spite of inactive Wnt signaling (Figure 1A–C). We found that an increase (2.2-fold) in the copy number of the *MYC* gene (Figure 6C) appeared to be responsible for the upregulation. A high degree (>2.0-fold) of *MYC* oncogene amplification is known to be infrequent in synovial sarcoma [26]. However, nuclear expression of c-MYC was detected in 85% (17/20) of clinical specimens and was frequent (≥30% of tumor cells) in 15% of them (3/20) (Figure S2 and Table S1). The Aska cell line carried the normal copy number (1.0-fold) of *MYC* (Figure 6C), and its level of c-MYC expression was lower than in other cell lines (Figure 6B). Knockdown of *MYC* gene expression by siRNA reduced the viability of HS-SY-II, SYO-1, and Yamato cells, but Aska cells were insensitive to silencing of *MYC* (Figure 6D) and NCB-0846 (Figure 3A), suggesting that NCB-0846 induces apoptotic cell death of synovial sarcoma at least partially through transcriptional suppression of *MYC*.

## 3. Discussion

Conventional cytotoxic chemotherapeutic agents including anthracycline, ifomide, and trabectedin have proven to be effective for the treatment of metastatic synovial sarcoma [27], but their usage and efficacy are often limited by the emergence of adverse events and drug resistance. Pazopanib is the first and only molecular therapeutic agent approved for the treatment of multiple histological subtypes of soft tissue sarcoma [28]. Pazopanib is a multi-tyrosine kinase inhibitor, and its main mode of action is believed to be inhibition of vascular endothelial growth factor receptor (VEGF)-mediated tumor angiogenesis [29]. The median survival of synovial sarcoma patients treated with pazopanib, however, was only 10.6 months, and the pazopanib treatment was associated with a high frequency of adverse events including hypertension, thrombocytopenia, and pneumothorax [28,30]. Early clinical trials of T lymphocytes genetically engineered to target the NY-ESO-1 cancer/testis antigen have yielded promising results [31,32], but this cancer immunotherapy is applicable only to patients with the human leukocyte antigen (HLA)-A*0201 or -A*0206 type as well as expression of NY-ESO-1 in tumors. Moreover, autologous lymphocyte cultivation is incurs significant costs and requires long-term discontinuation of ongoing treatment, potentially leading to fatal disease progression. Frizzled homolog 10 (FZD10) has attracted attention as a promising therapeutic target for synovial sarcoma [33], as its expression is limited to the cell membrane of synovial sarcoma and absent from vital organs [34]. A recent first-in-human clinical trial clarified the biodistribution, safety, and recommended dose of a radiolabeled humanized monoclonal antibody to FZD10 [35], but its efficacy has not been established.

Synovial sarcoma is uniquely characterized by the balanced chromosomal translocation t[X, 18; p11, q11], demonstrable in virtually all cases and not found in any other human neoplasms [2,8]. This translocation creates an in-frame fusion of SS18 to SSX1, SSX2, or SSX4, whereby all but the eight C-terminal amino acids of SS18 are replaced by the 78 C-terminal amino acids of the SSX partner. Kadoch and Crabtree observed that SS18-SSX was incorporated into the SWI/SNF (SWItch/Sucrose Non-Fermentable) complex [36]. Middeljans and colleagues reported that expression of the fusion oncogene induced depletion of the BAF47 (*SMARCB1*) subunit from the SWI/SNF complex [37]. Potential convergence may exist between the SWI/SNF complex and Wnt signaling, as loss of *SMARCB1* reportedly activates Wnt signaling [38]. Barham and colleagues [17] provided direct evidence for involvement of Wnt signaling in the SS18-SSX-mediated carcinogenesis of synovial sarcoma. The Wnt signaling pathway is aberrantly activated in an SS18-SSX2 transgenic mouse model, and genetic loss of β-catenin (*Ctnnb1*) blocks tumor formation in this model. Trautmann and colleagues [14] found that introduction of SS18-SSX into untransformed cells induced transactivation of Wnt target genes. Synovial sarcoma cell lines (SYO-1, CME-1, and HS-SY-II) showed sensitivity to three small-molecule inhibitors of the TCF/β-catenin complex (PKF115–584, CGP049090, and PKF118–310). β-Catenin stabilization in a transgenic animal model reportedly enhanced SS18-SSX-driven tumorigenesis and produced more dedifferentiated tumors [39]. Based on these findings, it is considered feasible to target a signaling molecule of the Wnt signaling pathway in synovial sarcoma.

TNIK is a component of the TCF4 and β-catenin transcriptional complex and functions as an essential co-regulator of Wnt target gene expression [19,40]. We screened a chemical library and identified a small-molecule TNIK-inhibitory compound named NCB-0846. This compound inhibited the expression of various Wnt target genes (such as *MYC*, *AXIN2*, and *CD44*) through conformational modification of TNIK and abrogated the stemness of colorectal cancer cells [21]. The *MYC* oncogene is a direct target of TCF/LEF family transcription factors [24] and centrally mediates the oncogenic activity of Wnt signaling [25]. In the present study, we revealed that Wnt signaling is activated in synovial sarcoma cells (Figure 1) and that siRNA-mediated or pharmacological TNIK inhibition reduced their viability and induced apoptosis (Figure 2 and Figure 3). NCB-0846 suppressed the expression of *MYC* and other Wnt target genes (Figure 5). Synovial sarcoma cell lines with high c-MYC protein expression were sensitive to the compound (Figure 3) and to the gene silencing of *MYC* (Figure 6). These results suggest that transcriptional *MYC* gene suppression is the central mode of action (MOA) of NCB-0846.

c-MYC is a versatile transcription factor that regulates the expression of genes involved in various biological functions such as cell proliferation, apoptosis, differentiation, and metabolism [41], and its inhibition would be expected to have a huge impact on the cancer transcriptome [42]. Aberrant expression or gene amplification of *MYC* has been implicated in the aggressiveness of various malignancies [43,44]. Shen and colleagues examined 32 cases of limb synovial sarcoma immunohistochemically and revealed a significant association of c-MYC expression with poor patient prognosis [45]. Synovial sarcoma is histologically divided into monophasic, biphasic, and poorly differentiated subtypes. We previously revealed the significant association of poorly differentiated synovial sarcoma with the expression of *MYC* [46]. Patients with poorly differentiated synovial sarcoma showed a high risk of recurrence [47]. NCB-0846 may be effective for the treatment of aggressive poorly differentiated synovial sarcoma.

In conclusion, we demonstrated for the first time that TNIK is a feasible drug target in synovial sarcoma. No effective molecular therapeutics have yet been approved for this lethal disease. We observed marked regression of xenografts even after the first oral administration of NCB-846, confirming its high efficacy. The compound is now under preclinical development aimed at investigational new drug (IND) application.

## 4. Materials and Methods

### 4.1. Ethical Issues

All of the animal experimental protocols in this study were reviewed and approved by the ethics and recombination safety committees of the National Cancer Center Research Institute (Tokyo, Japan) (T-17-022-m01, approved on 21 July 2017). The minimum number of animals necessary to obtain reliable results was used, and maximum attention was paid to animal rights and welfare protection. The use of human materials was reviewed and approved by the Institutional Review Board (IRB) of the National Cancer Center (Tokyo, Japan) (2004-050, approved on 30 October 2014 and revised on 7 November 2019). All patients gave their informed consent at the time. The IRB waived the requirement for obtaining new informed consent for this retrospective study. The investigations were carried out in accordance with the Declaration of Helsinki (https://www.wma.net/what-we-do/medical-ethics/declaration-of-helsinki/).

### 4.2. Cell Lines

Human synovial sarcoma HS-SY-II, Aska [48], and Yamato [48] cell lines were obtained from the Riken BioResource Center (Tsukuba, Japan). The SYO-1 cell line was established by one of the authors (A.K.) [49]. All cell lines were maintained in Dulbecco’s modified Eagle medium (Thermo Fisher Scientific, Waltham, MA, USA) supplemented with 10–20% fetal calf serum (Thermo Fisher Scientific). Absence of mycoplasma contamination was routinely confirmed using the e-Myco VALiD Mycoplasma PCR Detection Kit (iNtRon Biotechnology, Seoul, Korea).

### 4.3. Luciferase Reporter Assay

A pair of luciferase reporter constructs, super TOP-FLASH and super FOP-FLASH (Addgene, Watertown, MA, USA), was used to evaluate TCF/LEF transcriptional activity. Cells were transiently transfected in triplicate with one of the luciferase reporters and phRL-TK (Promega, Madison, WI, USA) (internal control) [18]. Luciferase activity was measured using the Dual-Luciferase Reporter Assay System (Promega) and normalized to that of *Renilla reniformis*. Data are presented as the ratio of TOP-FLASH to FOP-FLASH (TOP/FOP ratio).

### 4.4. Antibodies

Antibodies used in this study are listed in Table S4.

### 4.5. Immunoblot Analysis

Protein samples were fractionated by SDS–PAGE and blotted onto Immobilon-P membranes (Millipore, Burlington, MA, USA) as described previously [50]. After incubation with the primary antibodies at 4 °C overnight, the blots were detected with the relevant horseradish-peroxidase-conjugated anti-mouse or anti-rabbit IgG antibody (Cell Signaling Technology, Danvers, MA, USA) and Western lighting ECL Pro (PerkinElmer, Waltham, MA, USA). Signals were visualized with the LAS-4010 system (GE Healthcare, Chicago, IL, USA) and quantified using the ImageJ software package [51]. The uncropped images and relative quantification of blots in Figure 1B, Figure 2F, Figure 3E and Figure 6B are shown in Figures S3–S6, respectively.

### 4.6. Immunofluorescence Microscopy

Cells were fixed with 4% paraformaldehyde (PFA) for 10 min and permeabilized in 0.5% Triton X-100 for 3 min. The fixed cells were incubated with a primary antibody overnight at 4 °C and subsequently with a relevant secondary antibody (AlexaFluor 488-conjugated anti-rabbit IgG or AlexaFluor 568-conjugated anti-mouse IgG, Invitrogen, Waltham, MA, USA) for 1 h at 37 °C. The nuclei were stained with DAPI (Vectashield HardSet Mounting Medium with DAPI, Vector Laboratories, Burlingame, CA, USA). Images were captured using a TCS SP8 confocal microscope (Leica Microsystems, Wetzlar, Germany).

### 4.7. Patients and Tumor Samples

The study included tumor tissues surgically resected from 20 patients with synovial sarcoma (10 women and 10 men; median age at diagnosis 50 years, range 5–71 years). Clinicopathological characteristics are summarized in Table S1. The diagnosis of synovial sarcoma was made by a pathologist specialized in soft tissue sarcomas (A.Y.) and confirmed by the detection of *SS18* rearrangement by fluorescence in situ hybridization (FISH) or RT-PCR and/or the reduced expression of SMARCB1 [52,53].

### 4.8. Immunohistochemistry

Immunoperoxidase staining was performed using the Ventana DABMap detection kit and an automated slide stainer (Discovery XT, Ventana Medical Systems, Oro Valley, AZ, USA) [54]. The stained tissues were scored as strong positive (++, ≥30%), positive (+, <30%), or negative (−) according to the percentage of tumor cells with nuclear expression (Figure S7).

### 4.9. Gene Silencing by RNA Interference

Cells seeded at 50–70% confluency were transfected with siTNIK (s22905, s22906, and s22907; Thermo Fisher Scientific) and siMYC (s9129 and s9130; Thermo Fisher Scientific) at a final concentration of 50 nM in accordance with the manufacturer’s instructions.

### 4.10. Real-Time RT-PCR

Total RNA was prepared with a RNeasy Plus Mini Kit and treated with RNase-free DNase (Qiagen, Hilden, Germany). The cDNA was synthesized using a High-Capacity cDNA reverse transcription kit (Thermo Fisher Scientific) and subjected to TaqMan gene expression assay using pre-designed primer and probe sets (listed in Table S5). Amplification data measured as an increase in reporter fluorescence were collected using the StepOne™ Real-Time PCR System (Thermo Fisher Scientific). The relative mRNA expression level normalized to the internal control (human β-actin (*ACTB*) gene) was calculated using the comparative threshold cycle (CT) method [18]. Experiments were performed in triplicate and repeated at least two times. Wnt Signaling Targets RT^2^ Profiler PCR Arrays (Qiagen) were used for pathway-focused gene expression analyses.

### 4.11. Digital PCR

Total DNA was extracted from 5 × 10^6^ cells using the DNA Easy Blood and Tissue kit (Qiagen), in accordance with the manufacturer’s instructions. Copy number variation (CNV) data were obtained by the QuantStudio 3D digital PCR system (Life Technologies, Carlsbad, CA, USA) using pre-designed primer and probe sets (listed in Table S6) and analyzed with the QuantStudio 3D Analysis Suite Cloud software (Thermo Fisher Scientific). RNase P (*RPPH1*) was selected as an internal standard gene (Table S6).

### 4.12. Real-Time Cell Analysis (RTCA)

Cells were seeded at 5000 cells per well in 96 well clusters one day before transfection with control RNA (siCtrl) or siRNA to *TNIK* (siTNIK) or *MYC* (siMYC) using Lipofectamine RNAiMAX (Invitrogen). Cell growth was monitored periodically by a real-time cell electronic sensing analyzer (xCELLigence, ACEA Biosciences, Santa Clara, CA, USA) for 108 h via calculation of cell index (https://www.aceabio.com/products/icelligence/). Experiments were performed in triplicate and repeated two times.

### 4.13. Real-Time Monitoring of Transcriptional Activity

Lentiviral reporter gene transfer was used to evaluate the TCF/LEF transcriptional activity of HS-SY-II after transfection with siCtrl or siTNIK. Cells were infected with TCF/LEF reporter lentiviral particles encoding the GFP gene under control of the TCF/LEF-responsive promoter (Signal Lenti TCF/LEF Reporter (GFP) (Qiagen)) at a multiplicity of infection of 10 in the presence of 4 μg/mL SureEntry Transduction Reagent (Qiagen) for 24 h. GFP-positive cells were cloned by limiting dilution in the presence of 2 μg/mL puromycin (Sigma-Aldrich, St. Louis, MO, USA) and sorted with an S3e cell sorter (BIO-RAD, Hercules, CA, USA). The cells were seeded at a density of 20,000 per well in 96 well plates (Corning, Corning, NY, USA) and transfected with siCtrl or siTNIK. The amount of fluorescence was measured using Incucyte ZOOM (Essen BioScience, Tokyo, Japan).

### 4.14. Drug Sensitivity

Cells were seeded at a density of 3000 per well in 96 well plates. Twenty-four hours after seeding, the cells were exposed to serially diluted compounds (0.003, 0.01, 0.03, 0.1, 0.3, 1, 3, and 10 µM) and incubated for 72 h. ATP production was measured using a Cell Titer-Glo Luminescent Cell Viability Assay kit (Promega).

### 4.15. Xenografts

Five million HS-SY-II cells suspended in PBS containing 25% Matrigel (BD Biosciences, Franklin Lakes, NJ, USA) were inoculated into the subcutaneous tissues of 6 week old female NOD/SCID (NOD.CB17-Prkdcscid/J) mice. When the average tumor volume reached ~200 mm^3^, the mice were randomized according to tumor volume (five mice/group) and administered water (vehicle alone) or 80 mg/kg (body weight) NCB-0846 HCl (NCB-1055) dissolved in water by oral gavage twice a day in a 7 day schedule of 5 days on and 2 days off.

### 4.16. Real-Time Monitoring of Apoptosis Induction

The Real-Time-Glo™ Annexin V Apoptosis and Necrosis Assay reagent (Promega) was prepared as instructed in its technical manual and added to culture media at the beginning of drug treatment. Luciferase activity was measured every 2 h using the GloMax Discover System (Promega).

### 4.17. Cell Cycle Analysis

Cells were dissociated with Accutase, fixed with 70% EtOH at 4 °C, stained with Guava Cell Cycle reagent (Merck-Millipore, Burlington, MA, USA) in accordance with the manufacturer’s instructions, and analyzed using a Guava easy Cyte HT flow cytometer (Merck-Millipore). Cell doublets were eliminated by doublet discrimination gating. Data were analyzed using the FLOWJO version 10 software package (Treestar, Ashland, OR, USA).

### 4.18. RNA Sequencing

Total RNAs were extracted from HS-SY-II cells treated with 3 μM NCB-0846 or 3 μM NCB-0970 for 6 h. After confirming the absence of contamination with genomic DNA using a 2200 TapeStation (Agilent, Santa Clara, CA, USA), the TruSeq Stranded mRNA SamplePrep Kit was used to construct the sequencing library (Illumina, San Diego, CA, USA), and the libraries were sequenced using Illumina NovaSeq 6000 using a NovaSeq 6000 S4 Reagent Kit. Base calling was performed using the Illumina Basecall Software (bcl2fastq2 v2.20) with default parameters. Gene lists extracted from the transcriptome analyses were uploaded to the Database for Annotation, Visualization, and Integrated Discovery (DAVID) Bioinformatics database (https://david.ncifcrf.gov/), and the statistical significance of functional annotation was evaluated. The pathway analysis was performed by displaying the DAVID data on a pathway map of KEGG (Kyoto Encyclopedia of Genes and Genomes (http://www.genome.jp/kegg/). Clustering analysis was performed with MeV (http://mev.tm4.org). GSEA software was used to evaluate the statistical significance of pathway enrichment and to calculate the NES.

### 4.19. Statistical Analysis

All statistical analyses were performed using GraphPad Prism 8 (GraphPad, San Diego, CA, USA). Unless otherwise indicated, two-tailed Student’s *t*-tests of two groups assuming equal variances were used to calculate *p* values. Differences at *p* < 0.05 were considered significant.

## 5. Conclusions

Synovial sarcoma is highly dependent upon the expression of TNIK for cell proliferation and survival, and a small-molecule TNIK inhibitor NCB-0846 induced rapid apoptotic death of synovial sarcoma cells. This study demonstrated for the first time the therapeutic potential of TNIK inhibition in synovial sarcoma.

## Supplementary Materials

The following are available online at https://www.mdpi.com/2072-6694/12/5/1258/s1, Figure S1: Mapping of differentially expressed genes onto the signaling pathways regulating pluripotency of stem cells. Yellow boxes indicate genes downregulated (>2-fold) by NCB-0846, Figure S2: Immunohistochemical analysis of the c-MYC proteins in clinical specimens of synovial sarcoma. Representative cases with strong positive (++) and negative (−) nuclear c-MYC expression are shown. Scale bars: 100 µm in low-power views (left) and 20 µm in high-power views (right), Figure S3. Uncropped immunoblots of Figure 1B. The expression levels of axis inhibition protein 2 (AXIN2) were normalized to those of γ-tubulin, and quantification relative to HS-SY-II is shown below the blots, Figure S4. Uncropped immunoblots of Figure 2F. The expression levels of Traf2-and-Nck-interacting kinase (TNIK) and cleaved poly (ADP-ribose) polymerase-1 (PARP-1) were normalized to those of γ-tubulin, and quantification relative to siCtrl is shown below the blots, Figure S5. Uncropped immunoblots of Figure 3E. The expression levels of cleaved PARP-1 were normalized to those of γ-tubulin, and quantification relative to the dimethyl sulfoxide (DMSO) control is shown below the blots, Figure S6. Uncropped immunoblots of Figure 6B. The expression levels of c-MYC were normalized to those of γ-tubulin, and quantification relative to HS-SY-II is shown below the blots, Figure S7. Scoring of immunohistochemistry. The 20 tissue samples of synovial sarcoma were scored as strong positive (++, ≥30%), positive (+, <30%), or negative (−) according to the percentage of tumor cells with nuclear β-catenin (top), TNIK (middle), and c-MYC (bottom) expression. Scale bars: 20 μm, Table S1: Expression of the β-catenin, TNIK, and c-MYC proteins in clinical specimens, Table S2: Pathway analysis of genes regulated by NCB-0846, Table S3: Regulation of Wnt target genes by NCB-0846, Table S4: List of antibodies used in this study, Table S5: Pre-designed primer and probe sets used for real-time RT-PCR, Table S6: Pre-designed primer and probe sets used for digital PCR.
