# Supplementary material for: Feasibility of Targeting Traf2-and-Nck-Interacting Kinase in Synovial Sarcoma
Source: Cancers (Basel). 2020 May 16;12(5):1258. doi: 10.3390/cancers12051258 (PMC7281028; doi:10.3390/cancers12051258)
Supplement: Supplementary file 1 [file cancers-12-01258-s001.pdf]

Supplementary Materials

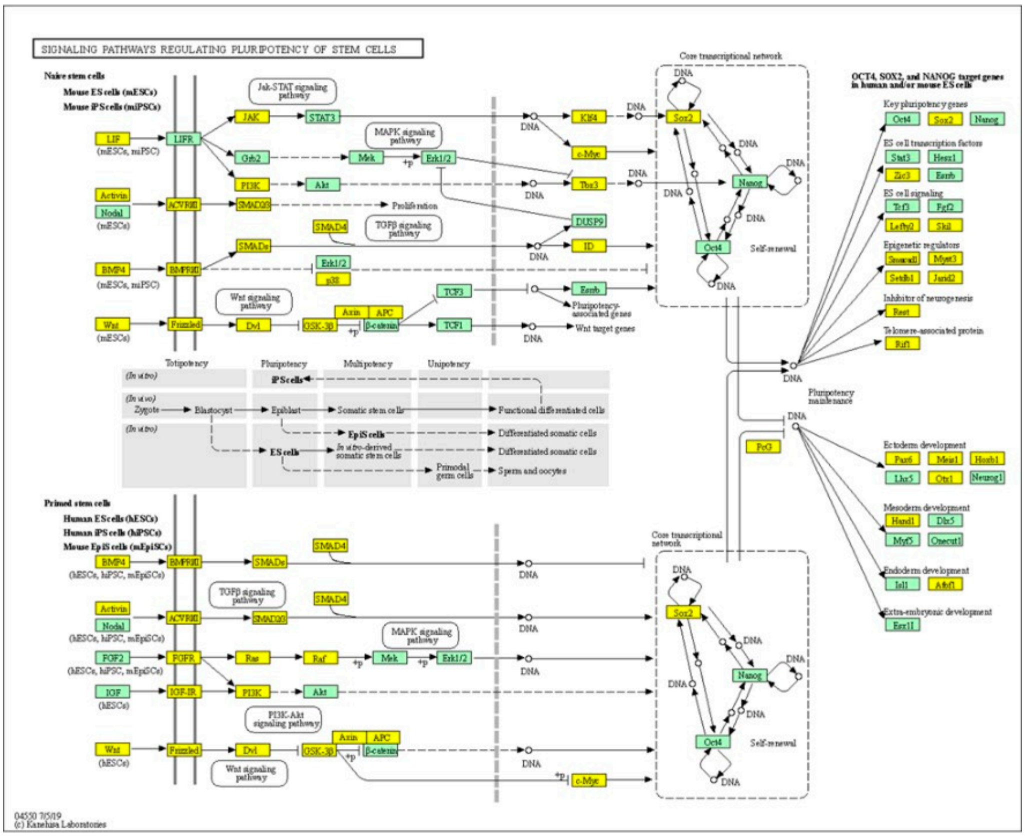

Figure S1. Mapping of differentially expressed genes onto the signaling pathways regulating pluripotency of stem cells. Yellow boxes indicate genes down-regulated (> 2 fold) by NCB-0846.

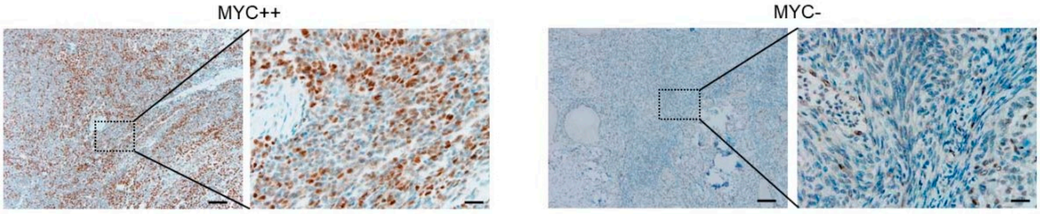

Figure S2. Immunohistochemical analysis of the c-MYC protein in clinical specimens of synovial sarcoma. Representative cases with strong positive (++) and negative (-) nuclear c-MYC expression are shown. Scale bars: 100 µm in low-power views (left) and 20 µm in high-power views (right).

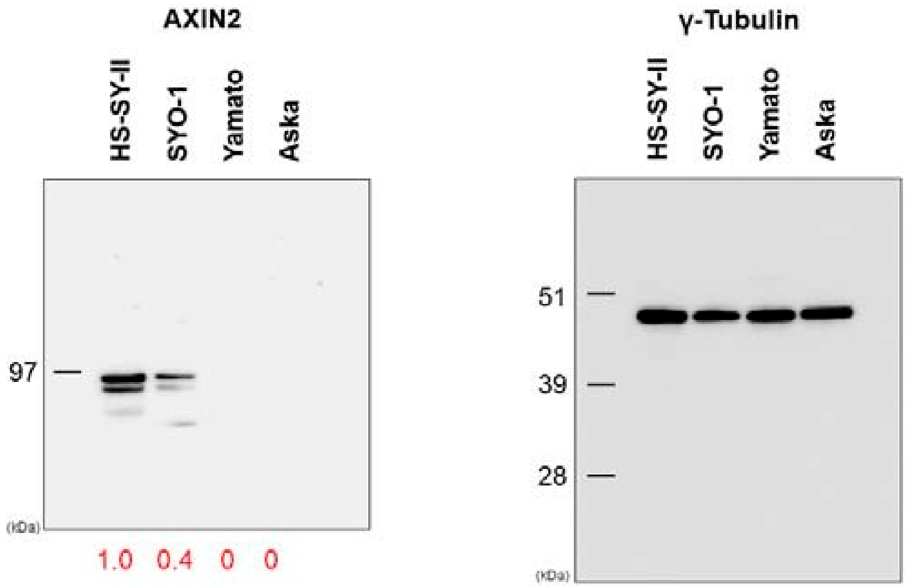

Figure S3. Uncropped immunoblots of Figure 1B. The expression levels of axis inhibition protein 2 (AXIN2) were normalized to those of γ-tubulin, and quantification relative to HS-SY-II is shown below the blots.

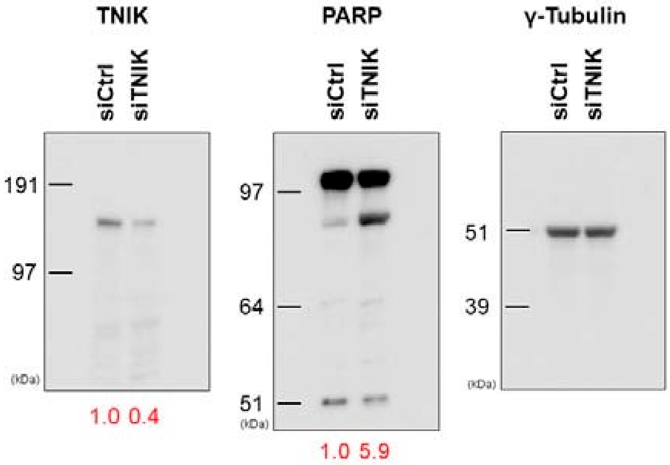

Figure S4. Uncropped immunoblots of Figure 2F. The expression levels of Traf2-and-Nck- interacting kinase (TNIK) and cleaved poly (ADP-ribose) polymerase-1 (PARP-1) were normalized to those of γ-tubulin, and quantification relative to siCtrl is shown below the blots.

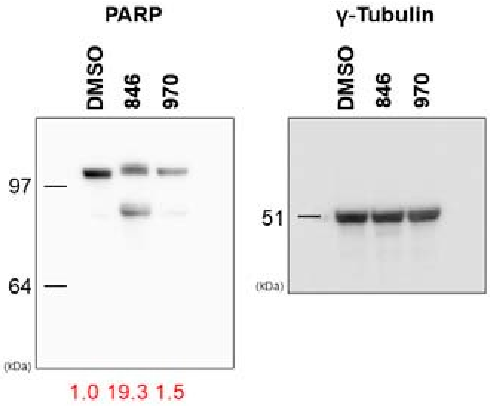

Figure S5. Uncropped immunoblots of Figure 3E. The expression levels of cleaved PARP-1 were normalized to those of γ-tubulin, and quantification relative to the dimethyl sulfoxide (DMSO) control is shown below the blots.

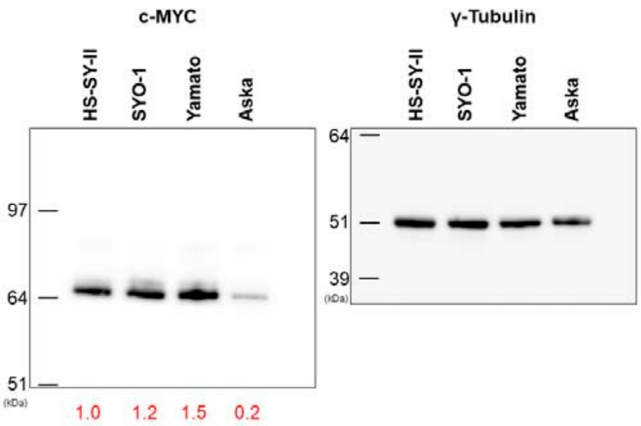

Figure S6. Uncropped immunoblots of Figure 6B. The expression levels of c-MYC were normalized to those of γ-tubulin, and quantification relative to HS-SY-II is shown below the blots.

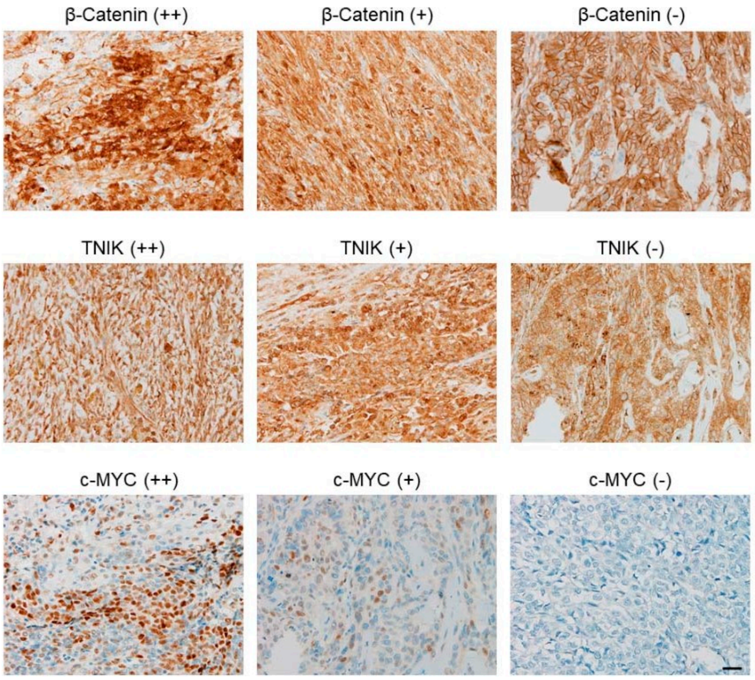

Figure S7. Scoring of immunohistochemistry. The 20 tissue samples of synovial sarcoma were scored as strong positive (++, ≥ 30%), positive (+, < 30%), or negative (−) according to the percentage of tumor cells with nuclear β-catenin (top), Traf2-and-Nck-interacting kinase (TNIK) (middle), and c-MYC (bottom) expression. Scale bars: 20 μm.

Table S1. Expression of the β-catenin, TNIK, and c-MYC proteins in clinical specimens.

Cas No.	Age (year)/Gender	Nuclear β-catenin	Nuclear TNIK	TNIK	c-MYC
1	52/F	+	+	+	−
2	54/M	+ +	+ +	+ +	−
3	57/F	+	+ +	+ +	+
4	29/F	+	+ +	+	+
5	48/F	+ +	+	+ +	+ +

6	52/F	−	−	+	−
7	54/M	+ +	+ +	+	+
8	46/F	+	+	+	+
9	22/M	+	+	+	+
10	51/F	+ +	+ +	+	+ +
11	56/M	+ +	+ +	+	+ +
12	20/F	−	−	+	+
13	58/M	+ +	+	+	+
14	34/M	+ +	+ +	+	+
15	71/F	+ +	+	+	+
16	45/M	+	+	+	+
17	60/M	+	+	+	+
18	19/F	+ +	+	+	−
19	23/M	+	+	+	+
20	5/M	+ +	+	+	+

Scored according to nuclear expression: −, negative; +, positive (< 30%); ++, strong positive (≥30%).

Table S2. Pathway analysis of genes regulated by NCB-0846.

	**Pathways**	**Genes**	***p*-value**	**Count**
1	Herpes simplex virus 1 infection	491	9×10^{-37}	333
2	Autophagy – animal	137	5×10^{-10}	91
3	Hippo signaling pathway	154	1×10^{-8}	97
4	MAPK signaling pathway	295	3×10^{-8}	165
5	Signaling pathways regulating pluripotency of stem cells	140	5×10^{-8}	88
6	Pathways in cancer	531	7×10^{-8}	273
7	Ubiquitin mediated proteolysis	136	1×10^{-7}	85
8	Chronic myeloid leukemia	76	2×10^{-7}	53
9	Colorectal cancer	86	3×10^{-7}	58
10	Neurotrophin signaling pathway	119	4×10^{-7}	75
11	Adherens junction	71	3×10^{-6}	48
12	TGF-β signaling pathway	94	3×10^{-6}	60
13	Wnt signaling pathway	160	4×10^{-6}	93
14	Pancreatic cancer	76	6×10^{-6}	50
15	Fanconi anemia pathway	54	8×10^{-6}	38
16	Axon guidance	181	9×10^{-6}	102
17	Endocytosis	249	1×10^{-5}	134
18	TNF signaling pathway	112	1×10^{-5}	68
19	Breast cancer	147	1×10^{-5}	85
20	FoxO signaling pathway	131	1×10^{-5}	77

Abbreviations: MAPK, mitogen-activated protein kinase; TGF, transforming growth factor; TNF, tumor necrosis factor; FoxO, forkhead box O.

Table S3. Regulation of Wnt target genes by NCB-0846.

	**NCB-0846**	**NCB-0970**	**846/970**	**846/970**
**Gene symbol**	**Average Ct**		**Delta CT**	**Fold change**
MYC	29.69	22.38	4.08	0.01
CUBN	40.00	33.40	1.49	0.01
AXIN2	28.82	23.17	2.78	0.02
GDF5	40.00	33.98	1.43	0.02
BMP4	32.15	26.85	1.77	0.03

VEGFA	31.57	26.45	1.80	0.03
DKK1	37.55	32.77	1.37	0.04
FZD7	28.24	23.54	2.33	0.04
PITX2	28.61	23.83	2.24	0.04
PTGS2	40.00	35.30	1.31	0.04
SOX2	31.61	27.13	1.63	0.04
SOX9	29.51	24.84	1.97	0.04
FGF9	32.57	28.31	1.51	0.05
ID2	26.91	22.63	2.63	0.05
POU5F1	33.25	28.92	1.49	0.05
BIRC5	31.92	27.92	1.51	0.06
FGF20	35.29	31.30	1.35	0.06
FOSL1	31.11	26.98	1.59	0.06
GDNF	33.35	29.41	1.42	0.06
IGF1	36.58	32.58	1.32	0.06
TFT	34.62	30.57	1.38	0.06
JAG1	28.20	24.47	1.84	0.07
AHR	36.40	32.70	1.29	0.08
BTRC	28.87	25.60	1.59	0.10
ETS2	29.13	25.88	1.55	0.10
PTCH1	34.25	30.89	1.31	0.10
DPP10	33.97	30.81	1.29	0.11
EFNB1	27.88	24.76	1.66	0.11
IL6	35.64	32.52	1.25	0.11
IRS1	29.96	26.96	1.43	0.12
KLF5	30.42	27.36	1.42	0.12
TWIST1	25.84	22.83	2.07	0.12
LRP1	28.39	25.60	1.50	0.14
WNT5A	26.26	23.40	1.84	0.14
FST	28.65	25.88	1.47	0.15
SFRP2	37.27	34.55	1.19	0.15
CACNA2D3	32.93	30.26	1.26	0.16
TLE1	26.57	23.92	1.68	0.16
WISP2	32.72	30.08	1.26	0.16
RUNX2	34.80	32.26	1.21	0.17
CEBPD	32.70	30.23	1.24	0.18
WISP1	34.51	32.07	1.20	0.18
CTGF	31.70	29.29	1.26	0.19
DAB2	28.20	25.84	1.41	0.19
CCND2	22.73	20.39	6.88	0.20
TCF4	25.00	22.69	1.86	0.20
WNT9A	32.49	30.27	1.22	0.21
CDON	30.21	28.02	1.27	0.22
MET	28.70	26.52	1.34	0.22
ANGPTL4	32.91	30.83	1.19	0.23
CDH1	33.67	31.57	1.18	0.23
FN1	30.21	28.09	1.26	0.23
TCF7L1	28.77	26.68	1.32	0.23
LEF1	25.99	23.96	1.52	0.24
SMO	27.69	25.66	1.36	0.24
NTRK2	32.27	30.30	1.19	0.25
PLAUR	29.65	27.68	1.26	0.25
CCND1	25.42	23.54	1.53	0.27
PPARD	28.38	26.54	1.28	0.28
TCF7L2	27.91	26.09	1.30	0.28
DLK1	28.37	26.59	1.27	0.29

MMP7	35.78	34.11	1.12	0.31
MMP9	34.38	32.78	1.13	0.33
TGFB3	29.93	28.34	1.19	0.33
B2M	24.26	22.66	1.61	0.33
WNT3A	31.54	29.99	1.16	0.34
GJA1	29.61	28.09	1.19	0.35
NRP1	25.78	24.29	1.35	0.35
CDKN2A	29.97	28.52	1.17	0.36
PDGFRA	27.87	26.42	1.23	0.36
NRCAM	35.18	33.82	1.10	0.38
HPRT1	25.70	24.45	1.29	0.42
SIX1	23.77	22.55	1.48	0.43
RPLP0	18.37	17.25	0.59	0.45
BGLAP	29.61	28.49	1.13	0.46
ANTXR1	27.41	26.45	1.15	0.51
MMP2	24.81	23.95	1.22	0.55
TCF7	28.81	28.17	1.08	0.63
ACTB	19.30	18.94	0.64	0.77
ABCB1	40.00	40.00	1.00	0.99
EGR1	40.00	40.00	1.00	0.99
FGF4	40.00	40.00	1.00	0.99
FGF7	40.00	40.00	1.00	0.99
IGF2	28.00	28.01	1.00	1.00
GAPDH	19.98	20.00	-	1.00
PPAP2B	29.70	29.90	0.98	1.14
CD44	34.92	35.16	0.98	1.17
EGFR	28.70	29.32	0.93	1.53
NANOG	32.50	40.00	0.63	179.24

Table S4. List of antibodies used in this study.

Figure	Application	Antigen	Company	Identification
1B	IB	AXIN2	CST	2151
		γ-Tubulin	Sigma	T6557
1C	IF	TNIK	Sigma	HPA012128
		β-Catenin	BD	610153
1D	IHC	TNIK	Sigma	HPA012128
		β-Catenin	BD	610153
2F	IB	TNIK	BD	612250
		PARP-1	CST	9542
		γ-Tubulin	Sigma	T6557
3E	IB	PARP-1	CST	9542
		γ-Tubulin	Sigma	T6557
6A	IB	c-MYC	Abcam	ab32072
		γ-Tubulin	Sigma	T6557

Abbreviations: IHC, immunohistochemistry; IF, immunofluorescence; IB, immunoblot; AXIN2, axis inhibition protein 2; TNIK, Traf2- and Nck-interacting Kinase; PARP-1, Poly (ADP-ribose) polymerase-1; CST, Cell Signaling Technology; BD, Becton, Dickinson and Company.

Table S5. Pre-designed primer and probe sets used for real-time RT-PCR.

Gene symbol	Species	Company	Identification
TNIK	Human	Applied Biosystems	Hs00323234_m1
MYC	Human	Applied Biosystems	Hs00153408_m1
ACTB	Human	Applied Biosystems	Hs99999903_m1

Table S6. Pre-designed primer and probe sets used for digital PCR.

Gene symbol	Species	Company	Identification
RPPH1	Human	Applied Biosystems	4401631
MYC	Human	Applied Biosystems	Hs02758348_cn
